# Self-Perceptions of Aging Among Dementia Caregivers: Evidence from the UK Protect Study

**DOI:** 10.1093/geroni/igab046.2315

**Published:** 2021-12-17

**Authors:** Shelbie Turner, Serena Sabatini, Helen Brooker, Anne Corbett, Adam Hampshire

**Affiliations:** 1 Oregon State University, Oregon State University, Oregon, United States; 2 University of Exeter, Exeter, England, United Kingdom; 3 Exeter, University of Exeter, England, United Kingdom; 4 University of Exeter, University of Exeter Medical School, England, United Kingdom; 5 Imperial College London, London, England, United Kingdom

## Abstract

Contact with older adults impact the perceptions people have towards their own aging self (Jarrott & Savla, 2015) and how they prepare for their own age-related change (Kornadt et al., 2015). Caregivers have close, intimate contact with older adults, yet no research explores how that contact may impact caregivers’ perceptions of their own aging. In this exploratory study, we compare perceptions of one’s own aging between current and previous formal caregivers, non-formal caregivers, and never-caregivers. We utilized data from 1978 informal caregivers, 247 formal caregivers, and 5586 never-caregivers of the 2019 wave of the UK Protect Study. We conducted ANCOVA tests to compare global levels of Awareness of Age-Related Change (AARC) gains and losses, AARC gains and losses specific to cognition, attitudes towards one’s own aging, and felt age across the three subgroups of participants with different caregiving roles. Omnibus results suggested that there were significant group differences (p<.05) in global levels of AARC gains and losses, AARC gains specific to cognition, and attitudes towards one’s own aging (p<.05) for female, but not male, caregivers. However, effect sizes were either small or negligible. Therefore, despite frequent contact with older adults, dementia caregivers may not have better or worse self-perceptions of aging than non-caregivers. Such findings may be reflective of intergenerational ambivalence, and future work should consider how the nature of the caregiving situation (i.e. relationship quality, intensity of the care, caregiver burden) shapes caregivers’ perceptions of their own aging, especially over time as caregivers navigate their own aging processes.

